# Invasive Species, Health, and Global History Afterword: The Disavowal of Human Agency

**DOI:** 10.1093/jhmas/jrae047

**Published:** 2024-12-11

**Authors:** Sabine Clarke

**Affiliations:** University of York, UK

**Keywords:** locusts, invasions, agency, food, health

## Abstract

The papers in this special issue explore the metaphorical realms that inform discourses on disruptive plants and animals. They explore how species movements in the twentieth century were framed and interpreted, and the medical, scientific, legal, and bureaucratic processes that turned a non-native or mobile species into a formally designated “invasive” one. In doing so, they allow insight into the mechanisms of disavowal, how some species were constructed as the cause of disease and ecological change, while others escaped censure.

In the period between 1940 and 1960, swarms of the desert locust filled the skies of Ethiopia, Eritrea, Somaliland, and Kenya on numerous occasions. British officials based in Kenya saw locust invasions in the Horn of Africa as a threat to military supply lines, food security, and post-war development.^1^ Time and time again, British officers struggled to stop locusts from crossing the northern Kenyan border and spreading across the colony, stripping fields of maize and wheat as they moved southwards and westwards.^2^

In the diaries and reports of officers who were based in the Northern Frontier Province (NFP) of Kenya, locust movements were portrayed as a transgression of the political border between Kenya and neighboring territories, such as Italian Somaliland. “In January 1954, about fifty desert locust swarms, some of them very large, invaded Kenya from the Somali Peninsula,” wrote one official.^3^ Swarms of locusts were described by colonial officials as invasions because they were understood to originate outside of Kenya’s borders, on the Somali coast and in Saudi Arabia. The military and security connotations of these invasions were clear for administrators who were charged both with keeping locusts at bay, and also restricting the movements of Somali clans who moved across the Kenyan border at will with their grazing herds, despite the efforts of officials to stop them.^4^ In the case of both locusts and people, free movement across the border served to exacerbate a sense of lack of control. They exposed the political weakness of the colonial state.

Locusts also presented a threat to the claim that British control produced benefits to the people of Kenya. Locusts undermined plans for agricultural development, projects that were directly and indirectly related to health. While a minority of development projects were explicitly concerned with producing food for local consumption, the British Colonial Office claimed that the expansion of cash crops was a key driver for improving living conditions across the Colonial Empire. Increased agricultural productivity was said to mean increased government revenues, and therefore better services, including infant clinics, rural dispensaries, and sanitation schemes. As Christos Lynteris and Jules Skotnes-Brown point out in the Introduction to this issue, the problem of species invasions could be constructed as a problem of health and welfare. British struggles to prevent species invasions implied the failure of the vision of development that was promoted after 1940 in which there was, supposedly, a new focus on improving the living conditions of colonized peoples.

The term “species invasion” normally refers to a species that has established itself in an environment that is not considered its natural one; it is a dislocating, foreign presence.^5^ The notion of species invasions is often said to have originated in the work of ecologists in the 1950s, although Lynteris and Skotnes-Brown make the crucial point in the Introduction that animal movements were linked to the spread of disease much earlier. Charles Elton is usually said to have been responsible for the idea that non-native species led to extinctions and biodiversity loss when they became established in a new habitat. This was because the new arrivals preyed upon, outcompeted, or hybridized with native species. Invading species were a destabilizing force as they had not evolved to be a part of the existing ecosystem in the way that native species had done. This meant that the necessary checks, such as predators, were not in place to maintain equilibrium. The idea that new species disrupted the natural order was linked to the concept of a balance of nature, or stable ecosystems, that were said to result from the co-evolution of plants and animals over long periods of time.^6^ The eradication of plants or animals that are seen to threaten native species is often presented by scientific and government bodies as an act of restoration. As Maddy Pearson’s article shows, this work is done on the basis that an older or more natural ecological order needs to be re-established.^7^

The notion of a species invasion as the arrival of an alien presence is not in fact a good description of the appearance of locusts in Kenya, despite contemporary British presentations of the problem in this way. The desert locust had spread across the Horn of Africa at periodic intervals for a very long time before Britain made a concerted attempt to curb its movements in the period after 1940. These movements were part of a natural cycle in which the insects erupted, swarmed, and flew great distances to eat and breed, before finally receding. The natural habitat of the desert locust exists, in fact, in a dual state; it is both limited and enormously wide ranging. While the idea of invasion resides upon the idea that the locust has crossed borders, those borders were impositions on the range of the locust. There were locust movements before there were borders; the “invasiveness” of the desert locust is a man-made construction. As Admire Mseba describes in this issue, the reference to invasion constructs the locust as a political problem, a problem that can only be resolved through international cooperation in controlling movements from one country to another.^8^ It also functions to indicate that the arrival of locusts is a problem that must be fought; the threat of invasion is paired with the ideal of suppression. While the presence of swarms of locusts cannot be described as “unnatural,” they are destructive, and their movements must be inhibited. Species invasions are a problem – that is the consensus, or so it may seem.^9^

Ecologists have come to question the assertion that species movements are universally destructive events.^10^ Recent work on biodiversity and species movements has shown that, with the exception of islands, invasive species are not necessarily the negative forces often portrayed. Long-term scientific studies, for example, can demonstrate that while novel species can produce a decline of other species at introduction, this is temporary. The longer-term history of the cane toad, introduced to Queensland, Australia, in 1935, illustrates this point. At first the extremely venomous toad produced a catastrophic decline in many native large predators such as snakes, lizards, and crocodiles. Substantial efforts were made to try and stop the spread of the toad to avert what some called an “ecological Armageddon.” Mass extinctions did not in fact transpire in the end, as native species adapted, either by changing their behavior to avoid the toad, or avoid ingesting its most poisonous parts, and also by developing tolerance to its toxin.^11^ In addition to this example, scientists have looked at other cases to argue that rather than reducing biodiversity, non-native species can increase it overall.^12^ Invasive plant species such as the rhododendron, introduced to Britain in the nineteenth century, have been found to provide novel habitats for animals.^13^

For the historian, this reappraisal of the consequences of novel species movements is important. It is a good reminder of the care that needs to be taken when using organizing concepts from the sciences. While the adoption of ideas from ecology by environmental historians has been important and fruitful, there is the issue of how historians might use concepts that are the subject of debate and reassessment by their colleagues in the sciences. Deploying a concept or a definition from ecology as if it refers to a matter that is settled can leave historians open to the accusation that they are actively eliding scientific debate and instead using scientific terms merely to give some credence to their preferred analysis.^14^ This could have consequences for the credibility of fields such as environmental history.

The fact that some of the basic tenets of invasion biology are currently undergoing reassessment is a prompt for historical attention. As this special issue shows, the question that historians are equipped to answer is how and why did actors come to speak of the presence of certain species as dangerous for health and community wellbeing? What can we learn from examining the ways that species movements have been framed and interpreted? What or who was elided when foreign species were seen as the sole, or main, cause of disease and ecological change? What medical, scientific, legal, and bureaucratic processes turn a non-native or mobile species into a formally designated “invasive” one?^15^

These articles provide important insights into the ways in which invasive species have been characterized, exploring the metaphorical realms that inform discourses on disruptive plants and animals.^16^ Kenneth Reilly, for example, shows just how significant a wider field of association can be in constructing a species as a threat, in his study of the connections made between crime and the kudzu vine in Atlanta. The papers in this special issue show that how we describe non-native or irruptive species often reveals more about us. As noted in many of the articles, so-called invaders have often been described in terms that are explicitly xenophobic and racist.^17^ As Lynteris, Skotnes-Brown and Mattheus Silva show, discussions of rats, gerbils and plague could invoke ideas of hygiene or pollution in which the alien other was a carrier of disease or corruption.^18^

Peter Coates tells how our representations of immigrants and alien species are shaped by a language of “exclusion and belonging.”^19^ Distinguishing between foreign and native species contributes to our sense of identity through a nostalgic attachment to what some people consider the natural or traditional environment of their country. Jeannie Shinozuka has described how the hardening of attitudes towards immigrants, plants, and animals from Asia arose in tandem from the late nineteenth century onwards.^20^ She shows the traffic that occurred between the development of border systems involving differentiation, surveillance, and control that regulated the movement of people and those that regulated the movement of plants and animals.

Non-native plants and animals are not portrayed as problematic simply because their presence has unintended consequences for existing species or human health. Medical or legal discourse, for example, frames a species as problematic through the ways that these texts afford a plant or animal a particular type of agency. This is not just the notion that a species such as the gerbil, rat, or mink manifests a desire for new territory and therefore embodies cunning and opportunism, but also that they are unruly, out-of-control agents. This aspect of the imagined behavior of invasive species often involves a focus on their supposed over-fecundity, excessive appetite, or hyper-aggression. We reject Japanese knotweed, kudzu, mink, and sea lamprey not just on the basis that they are foreign invaders that might threaten familiar domestic species, or bring disease, but also for their lack of restraint – they consume too much, and they breed too much.^21^ These are species that do not know moderation, thus rhyming with the category of “vermin” as discussed by early modern historians.^22^ Certain plants and animals are destabilizing therefore, not because they are out of place, but because they are without temperance; they are morally wrong.^23^ Writers about species movements express not just intellectual opposition to some species as they upset the natural order, but also express active dislike of these species, communicating feelings of disapproval and even disgust. The similarities here with the ways in which immigrant communities are described form part of broader discursive configurations discussed in the Introduction of this special issue. A focus on the fecundity of a species such as the grey squirrel connects to the way that the issue of population growth in the Global South was portrayed in the mid-twentieth century. It brings to mind the notorious scene with which Paul Ehrlich chose to open *The Population Bomb* in which he claimed that the moment when the true magnitude of the population problem first hit him was during a visit to India when he was sitting in a taxi, “hopping with fleas” looking out on a slum where, “The streets seemed alive with people…People, people, people, people.”^24^

In the case of locusts, the focus on their supposed voracious appetite, the enormous size of their flying swarms, and the way that their eggs covered large areas, are all frequently mentioned by observers. These features of the behavior of the desert locust can be said to be rooted in observation, but when locust control officers and government officials spoke of the devastation wrought by plagues of locusts, they did not acknowledge the role that human behavior had in this.^25^ The focus on the extremes of the behavior of the insect – its insatiable character, the sheer scale of its mass swarming, breeding, and feeding – allowed the disavowal of human agency in producing a crisis in food production. The fact is that European cultivation in East Africa, particularly the crops raised by European settlers, allowed locust invasions to be larger and longer. The famous locust scientist Boris Uvarov speculated in 1949 that the frequency and intensity of locust invasions had increased over the course of the twentieth century, in line with the expansion of British agricultural development schemes.^26^ Human agency, as well as locust agency, had a role to play in producing the invasions between 1940 and 1960, but the overwhelming focus on the rapacious character of the insect allowed the role of people, and the significance of colonization, to be marginalized.

The papers in this special issue show us how an examination of the ways in which animal and plant invaders were spoken of in the past is important for what they might tell us about the anxieties of the time. Writing about the desert locust from the period between 1940 and 1960 is rich terrain for historians of Britain’s colonial empire, not just for what is said about how locust invasions occurred and were dealt with in practical terms, but also for revealing the ways that individuals might be seen to be trying to reconcile themselves to the failure or limitations of British power. There are in fact two narratives on locusts that can found in the archive. One celebrates the creation of new apparatus for effective desert locust control campaigns during the war and afterwards; this is the official version that made its way into reports and newspapers.^27^ The other narrative, a more personal one that did not become incorporated in the official version of events, shows how locust movements signified for some officers the ways in which their grip on the territory that they were charged with overseeing was so very tenuous.

Locusts were one repository for fears about the precarious or limited nature of colonial power. The problem was in part the challenge of managing the locust threat in places beyond the Kenya border in lands where Britain did not have formal control. Boris Uvarov’s Anti-Locust Research Centre in London had determined that control of “outbreak areas” of the desert locust was the only effective way to prevent invasions of Britain’s East African territories. The difficulty was that these outbreak areas were in places such as Pakistan, Saudi Arabia, and Oman. British negotiations with Saudi Arabia to gain access for British locust officers offered multiple opportunities for Britain to be confronted with the limits of its power in the world after 1945.^28^

The other issue was the fact that British officers based in Kenya, in the Northern Frontier Province (NFP) especially, had to face the fact that they could not master the environment they were charged with overseeing; they had neither the knowledge nor the means. Locust control required constant surveillance of a large and difficult landscape, without adequate roads and sometimes even maps. British officers were often reliant upon local people and their knowledge, employing them to work as locust scouts and as guides to the terrain.^29^ The fact that African peoples were more adept at navigating the environment of the Northern Frontier than the British was a problem. People evaded scrutiny and taxes, locust scouts took pay without doing their work, and there was the constant threat of sedition. A sense that the NFP was an ungovernable place is evident in the writings of the British staff stationed there. The non-official narrative of rule in the NFP was of attempts to police a border against the transgressions of locusts and people whose connection to the land was older and deeper than European colonizers. Uncontrolled movements across the board exposed again and again the fragile nature of claims to colonial control and the potential for so-called development. When the British deployed the idea of locust invasions, or tribal trespass, the issue was not that locusts or Somali people were alien to this part of the Kenyan environment. Locusts and African peoples had travelled various routes across the northern borders of Kenya for far longer than the British had been in East Africa. If there were invaders or aliens present in Kenya, then it was, of course, British colonists who were a people out-of-place.

## Footnotes

1 United Kingdom National Archives (UKNA), CO 927/154/5, Anti Locust Research Centre, Boris Uvarov, “Locust Research and Control, 1929-1949.”

2 See for example, National Archives of Kenya (KNA), East African High Commission, “Report of the Desert Locust Survey, 1^st^ October 1948-30^th^ September 1950.”

3 KNA, AR /16/12 Agricultural Department, Pest – Locust. 1954.

4 KNA, DC/WAJ/2/6/21, 1953, handover report from G.S. Snell to J.M. Elliott.

5 Charles Elton is credited with founding invasion ecology with his book, *The Ecology of Invasions by Animals and Plants* (London: Methuen, 1958).

6 Ibid.

7 See Maddy Pearson, “Furry, Feral, Foe,” this issue.

8 See Admire Mseba, “Politics, Technoscience and the Environment,” and also Jules Skotnes-Brown and Matheus Alves Duarte, “Gerbils without Borders,” this issue.

9 Elton, *Ecology of Invasions by Animals and Plants*.

10 For a good summary of work by ecologists that is critical of invasion biology, see Fred Pearce, *The New Wild: Why Invasive Species will be Nature’s Salvation* (London: Ikon Books, 2016); Chris D. Thomas, *Inheritors of the Earth: How Nature is Thriving in the Age of Extinction* (London: Penguin, 2018).

11 Richard Shine, “The Ecological Impact of Invasive Cane Toads (*Bufo marinus*) in Australia,” *Quarterly Review of Biology* 85 (2010): 253-291.

12 Chris D. Thomas, “The Anthropocene Could Raise Biological Diversity,” *Nature* 502 (2013): 7.

13 Aurelio F. Malo, Ben Godsall, Clare Prebble, Zoe Grange, Samantha McCandless, Andrew Taylor, Tim Coulson, “Positive Effects of an Invasive Shrub on Aggregation and Abundance of a Native Small Rodent,” *Behavioral Ecology* 24 (2013): 759-767.

14 Apart from invasion biology, see the concept of the “Insect Apocalypse” or “Insectageddon.” A good summary of some of the issues that relate to this concept can be found in Chris D. Thomas, T. Hefin Jones, and Sue E. Hartley, “‘Insectageddon’; A Call for More Robust Data and Rigorous Analyses,” *Global Change Biology* 25 (2019): 1891-1892. The point is not that there is, or is not, decline in insects, but more that recommendations for action need to be based on good evidence and at present the evidence is too partial and sometimes too selective.

15 Vincent Bijman, “The Sea Lamprey Controversy,” this issue.

16 See Christos Lynteris, “A Rat’s Progress,” this issue.

17 Peter Coates, *American Perceptions of Immigrant and Invasive Species: Strangers on the Land* (Berkeley: University of California Press, 2006).

18 See Lynteris, “A Rat’s Progress” and Jules Skotnes-Brown and Matheus Alves Duarte, “Gerbils without Borders,” this issue.

19 Coates, *American Perceptions of Immigrant and Invasive Species*.

20 Jennie N. Shinozuka, *Biotic Borders. Transpacific Plant and Insect Migration and the Rise of Anti-Asian Racism in America, 1890-1950* (Chicago: University of Chicago Press, 2022).

21 See for example, Kenneth Reilly, “Covering For Our City Blight” and Pearson,” Furry, Feral, Foe,” this issue.

22 Mary Fissell, “Imagining Vermin in Early Modern England,” *History Workshop Journal* 47 (1999): 1–29; Lucinda Cole, *Imperfect Creatures: Vermin, Literature, and the Sciences of Life, 1600–1740* (Ann Arbor: University of Michigan Press, 2016).

23 There are echoes here with ways in which “tropicality” conveyed the idea of the tropics as a place of excess, in opposition to the moral virtue associated with more temperate regions of the world. Nancy Leys Stepan, *Picturing Tropical Nature* (London: Reaktion Books, 2001).

24 Paul Ehrlich, *The Population Bomb* (Sierra Club: Ballantine Books, 1968), 1.

25 See also Mseba, “Politics, Technoscience and the Environment,” this issue.

26 UKNA, CO 927/154/5, Boris Uvarov, “Locust Research and Control, 1929-1949.”

27 For a good summary of British achievements in locust control see, Michael Worboys, “Imperial Entomology: Boris P. Uvarov and Locusts, c.1920-1950,” *British Journal for the History of Science* 55 (2022): 27-51.

28 KNA, BV/3/187, “Desert Locust Survey, Minutes of meetings, 50^th^ Meeting DLC Executive Committee, 8/1/53.”

29 KNA, RS/8/1, Desert Locust Survey, Meeting of the DLS Advisory Committee, Nairobi, 1952; KNA, RS/8/1, Locust campaigns, complaints, 1944/5.

